# Opsoclonus-myoclonus-ataxia syndrome in an AIDS patient

**DOI:** 10.1590/S1679-45082013000400023

**Published:** 2013

**Authors:** Thiago Cardoso Vale, Rodrigo Alencar e Silva, Mauro César Quintão e Silva Cunningham, Débora Palma Maia, Sarah Teixeira Camargos, Francisco Cardoso

**Affiliations:** 1 Universidade Federal de Juiz de Fora, Juiz de Fora, MG, Brazil.; 2 Universidade Federal de Minas Gerais, Belo Horizonte, MG, Brazil.

**Keywords:** Ocular motility disorders, Ataxia, Myoclonus, Ataxic gait, HIV

## Abstract

We report the case of a 38-year-old woman with AIDS who developed opsoclonus-myoclonus-ataxia syndrome during a period different from other cases reported in literature. Opsoclonus-myoclonus-ataxia syndrome had already been reported as the initial neurological presentation of AIDS, as well as at the time of HIV-seroconversion and immune reconstitution syndrome. Our case is unique since the patient had an elevated CD4 count and negative viral load in the period when the opsoclonus-myoclonus-ataxia syndrome occurred.

## RESUMO

É relatado aqui o caso de uma mulher de 38 anos com AIDS que desenvolveu a síndrome de opsoclonia-mioclonia-ataxia em um período diferente dos outros casos já relatados na literatura. A síndrome de opsoclonia-mioclonia-ataxia já tinha sido relatada como manifestação inicial de AIDS, assim como no momento da soroconversão de HIV e na síndrome de reconstituição imune. Este caso é único, uma vez que a paciente tinha contagem elevada de CD4 e carga viral negativa no momento em que a síndrome de opsoclonia-mioclonia-ataxia ocorreu.

## INTRODUCTION

Opsoclonus-myoclonus-ataxia syndrome (OMAS) is characterized by continuous multidirectional saccadic eye movements accompanied by generalized myoclonus and, less frequently, cerebellar ataxia, postural tremor, encephalopathy, and behavioral disturbances. It is also known as “dancing eye and dancing feet syndrome”^(1)^. Although most cases of OMAS are paraneoplastic (typically associated with breast and small cell lung cancers), toxic-metabolic, or idiopathic, some are post-infectious. There have been reports of this syndrome in patients with AIDS after seroconversion and as part of an immune reconstitution syndrome^(2)^. To the best of our knowledge, we present the first case of OMAS in a patient with AIDS without criteria for both previous scenarios of the course of the infection.

## CASE REPORT

**Figure 1:**
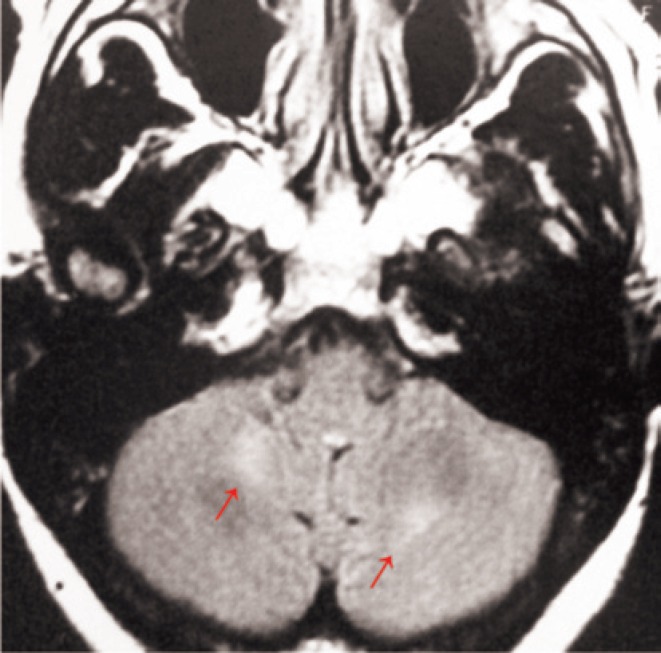
Contrasted T1-weighted magnetic resonance imaging sequence showing nonspecific hyperintensities in cerebellar hemispheres (red arrows)

A 38-year-old woman had a history of schizophrenia for the past 16 years and AIDS since she was 30 years old. She was on haloperidol and levomepromazine since the psychiatric diagnosis, as well as highly active antiretroviral therapy (HAART) comprised of lamivudine, tenofovir, and ritonavir-boosted atazanavir. She was diagnosed as AIDS patient in 2002 after presenting with recurrent pneumonia, sinusitis, and two episodes of herpes-zoster infection. In 2004, she received the first HAART scheme after a CD4+ count of only 39 cells/mm^3^. Initially, the patient did not comply with treatment, resulting in a CD4+ count of 13 cells/mm^3^ in 2005 and prompting a new HAART scheme. Her CD4+ count gradually raised and resulted in a negative viral load at the beginning of 2006. In April 2010, with a CD4+ count of 537 cells/mm^3^ and a negative viral load, the patient presented with vomiting, headache, a wide-base unsteady gait, bradykinesia, rigidity, facial hypomimia and mild uncontrolled jerky movements of the eyes, head, and fingers of both hands. She was diagnosed with OMAS and drug-induced parkinsonian syndrome. Cranial computed tomography was normal and brain magnetic resonance imaging revealed nonspecific white matter abnormalities in periventricular areas, cerebellar hemispheres, left temporal lobe, and thalamus (Figure 1). Cerebrospinal fluid (CSF) analysis showed lymphocytic pleocytosis (22 leukocytes/mm^3^ with 95% of lymphocytes), 43mg/dL of proteins, and 49mg/dL of glucose. CSF *Gram* stain, cultures, and VDRL were negative. Latex-cryptococcus antigen test and polymerase chain reaction (PCR) for tuberculosis and herpes-simplex virus were negative. Serum protozoan and viral IgM serologies (toxoplasmosis, cytomegalovirus, and herpes-simplex virus) were also negative. She was treated with clonazepam with gradual improvement of vomiting, headache, ataxia, and opsoclonus, but persistence of distal myoclonus in the upper limbs.

## DISCUSSION

There are less than a handful of case reports on OMAS occurring in HIV-patients^(3–5)^. All cases were reported in four different stages of HIV-infection: three cases as the initial neurological presentation, two cases at the time of HIV-seroconversion, two cases as part of the immune reconstitution syndrome, and one case at the time of tuberculosis reactivation. Our case is unique since the patient had elevated CD4 count and negative viral load in the period when OMAS occurred.

The mechanism underlying the pathogenesis of OMAS remains to be determined. Notwithstanding, robust evidence supports an autoimmune basis for OMAS affecting the brainstem, the pontine paramedian reticular formation, and cerebellar circuits^(1,4)^. A humoral immune mechanism could be suggested by the response of OMAS to immune therapy in a few patients and by the detection of autoantibodies. Zandman-Goddard et al.^(5)^ showed that patients with HIV with stable CD4+ counts are more prone to having autoimmune conditions than the general population, and often have a range of autoantibodies even when asymptomatic. On the other hand, due to absence of specific autoantibodies in all OMAS cases and the inability to passively transfer OMAS to animals, involvement of humoral immunity is less probable, suggesting the role of cell-mediated immunity. HIV-associated OMAS may be the consequence of an imbalance inflammation in which a reduced CD4/CD8 ratio, in addition to a critical level of functional CD4+ cells for efficient CD8+ cytotoxicity, results in dysfunction of brainstem-cerebellar circuitry in susceptible individuals^(3)^. OMAS may improve spontaneously when mild, or require immune therapy when symptoms are persistent. Due to the difficulties of using immunosuppressive therapy in HIV+ patients and the lesser severity of her OMAS, she was treated with benzodiazepines. The patient improved rather quickly, but persisted with distal myoclonus.

## CONCLUSION

This is a case report of an AIDS patient who developed OMAS in a particular period of the infection not previously reported in the literature.
